# Lithium and Erectile Dysfunction: An Overview

**DOI:** 10.3390/cells11010171

**Published:** 2022-01-05

**Authors:** Mohammad Sheibani, Mehdi Ghasemi, Ahmad Reza Dehpour

**Affiliations:** 1 Department of Pharmacology, School of Medicine, Iran University of Medical Sciences, Tehran 14496-14535, Iran; mohammad.sheibani89@gmail.com; 2 Department of Neurology, University of Massachusetts Chan Medical School, Worcester, MA 01655, USA; 3 Department of Pharmacology, School of Medicine, Tehran University of Medical Sciences, Tehran 14176-13151, Iran; 4 Experimental Medicine Research Center, School of Medicine, Tehran University of Medical Sciences, Tehran 14176-13151, Iran

**Keywords:** lithium, sexual dysfunction, erectile dysfunction, corpus cavernosum, nitric oxide, nitric oxide synthase

## Abstract

Lithium has been a mainstay of therapy for patients with bipolar disorders for several decades. However, it may exert a variety of adverse effects that can affect patients’ compliance. Sexual and erectile dysfunction has been reported in several studies by patients who take lithium as monotherapy or combined with other psychotherapeutic agents. The exact mechanisms underlying such side effects of lithium are not completely understood. It seems that both central and peripheral mechanisms are involved in the lithium-related sexual dysfunction. Here, we had an overview of the epidemiology of lithium-related sexual and erectile dysfunction in previous clinical studies as well as possible pathologic pathways that could be involved in this adverse effect of lithium based on the previous preclinical studies. Understanding such mechanisms could potentially open a new avenue for therapies that can overcome lithium-related sexual dysfunction and improve patients’ adherence to the medication intake.

## 1. Introduction

Lithium is a monovalent cation (Li^+^) when it loses the only electron of the second orbital. Its biological importance is based on the therapeutic properties of its salts in the management of psychiatric diseases, including mood and psychotic disorders [1,2], which was first described by the Australian psychiatrist John F. Cade in 1949 [3]. These days, there is no doubt that this agent is effective in the management of bipolar disorders [4] and can reduce both suicide and mortality in mood disorders [5,6]. In recent years, multiple molecular mechanisms related to the therapeutic properties of lithium have been recognized; however, no definitive mechanism for its therapeutic effects has been established [7]. Lithium could have gene regulatory function through affecting nuclear receptors, and subsequently the regulation of the expression of various central neurotransmitters or their receptors [7,8]. For example, it may enhance the neuronal release of serotonin in the raphe nucleus during depolarization [9]. Moreover, lithium inhibits the glycogen synthase kinase 3 β (GSK3β) that phosphorylates Rev-Erbα (an intracellular transcription factor protein), and as a result, inhibits the circadian clock. This will disturb many biological functions governed by the brain, such as metabolism, sleep (diurnal rhythm), and body temperature [10,11]. Pre-clinical studies have also demonstrated a crucial role for *N*-methyl-D-aspartate (NMDA) receptor/nitric oxide (NO) signaling pathways, which play important roles in neural plasticity (brain plasticity) and in the antidepressant effects of lithium [7,12,13,14]. Disturbance in the inositol pathway has been associated with depression and memory impairment. Lithium inhibits the enzyme inositol monophosphates [15]. This enzyme dephosphorylates inositol monophosphate to free inositol, and as a result, the inositol pool is attenuated. This could then clarify the therapeutic function of lithium with slight effects on physiological behavior [16,17]. One suggested hypothesis is that the pathophysiology of bipolar disorders is linked to the super sensitivity of catecholamine receptors, and lithium interacts with cyclic adenosine monophosphate (cAMP)-mediated cascades and blocks the supersensitive catecholamine receptors of the neurons of the central nervous system (CNS) [18].

Despite its beneficial effects in mood and psychotic disorders, lithium may also exert a wide variety of adverse effects which are typically dose-related and include nervous, cardiac, renal, immune, metabolic, and endocrine abnormalities [19]. Like many other antipsychotic and antidepressant drugs, lithium may also cause sexual and erectile dysfunction [20,21]. About one-third of bipolar or schizoaffective patients receiving lithium report sexual dysfunction [20,21]. However, the exact mechanisms underlying such adverse effects of lithium have not been completely understood. This is an important consideration, as medications’ adverse effects (e.g., sexual dysfunction) is one of the major reasons for medication’s non-compliance as well as the negative impact on the quality of life of psychiatric patients. In this review, we will discuss the epidemiology of sexual and erectile dysfunction related to lithium intake, possible both central and peripheral mechanisms underlying such effects of lithium, and therapeutic approaches to potentially overcome these adverse effects of lithium.

## 2. Neuroanatomy and Physiology of Erectile Function

**Figure 1:**
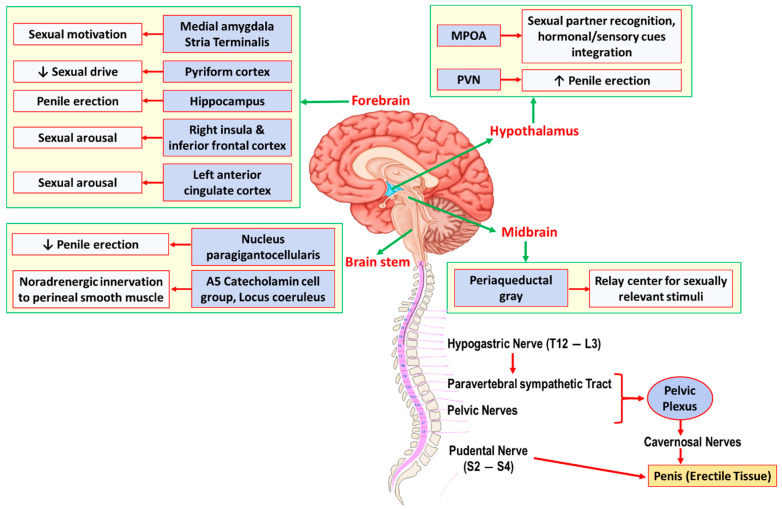
Schematic representation of central and peripheral neural pathways controlling the penile erection. Several brain regions including forebrain, midbrain, hypothalamus, and brainstem are involved in sexual drive, arousal, and ultimately erectile function. Sexual stimuli activate brain regions through which they stimulate the hypothalamus and its nuclei (mainly medial preoptic area [MPOA] and paraventricular nucleus [PVN]). The neural pathway then traverses through the medulla oblongata and the spinal cord to the genital apparatus, i.e., penile tissue in males. Two major nerves innervating the penis include (i) the Pudental nerve, which arises from sacral S2–S4 roots and contains the primary afferent sensory and efferent motor pathway to the penis, and (ii) the Cavernosal nerves, which contain the primary efferent sympathetic and parasympathetic pathways originating from the pelvic plexuses. Three nerve groups also innervate pelvic plexuses: (i) the hypogastric nerve (from T12–L3 nerve roots), (ii) pelvic nerves (from sacral nerve roots), and (iii) the post-ganglionic fibers from the paravertebral sympathetic thoracolumbar (T12–L3 levels) ganglia chain.

Erectile function is a complex neural central and peripheral interaction that involves several brain regions, the spinal cord, peripheral nerves, as well as the penile tissue (Figure 1). The hypothalamus (especially, the medial preoptic area [MPOA] and the paraventricular nucleus of the hypothalamus [PVN]) and hippocampus are the two most important integration centers for sexual function and penile erection [22,23]. Dopaminergic efferent pathways from the MPOA to the medial forebrain bundle and the midbrain tegmental region (near the substantia nigra) facilitate sexual behavior and penile erection [24,25]. Projecting premotor neurons from the PVN directly onto spinal autonomic preganglionic neurons also play essential roles in penile erection. These neurons contain various neurotransmitters, including oxytocin, vasopressin, glutamate, nitric oxide (NO), enkephalins, and dopamine [25]. Overall, an injection of oxytocin, vasopressin, glutamate, dopamine, and nitric oxide (NO) into the PVN elicits episodes of penile erection; however, endorphins and γ-aminobutyric acid B (GABA_B_) agonists have inhibitory effects [24,26]. Several brain stem and medullary regions are also involved in sexual function (Figure 1). The A5 catecholamine cell group in the pons and medulla, and locus coeruleus, have adrenergic innervation to the hypothalamus, thalamus, neocortex, and spinal cord. Serotonergic projections from the nucleus paragigantocellularis onto the hypothalamus, the limbic system, the neocortex, and the spinal cord have inhibitory effects on penile erection [27]. The central neural pathways for erectile function overall traverses through the medulla oblongata and the spinal cord to the penile tissue. Two major nerves innervating the penis include the (i) pudental nerve, which arises from sacral S2–S4 roots and contains the primary afferent sensory and efferent motor pathway to the penis, and (ii) cavernosal nerves, which contain the primary efferent sympathetic and parasympathetic pathways originating from the pelvic plexuses. Three nerve groups also innervate pelvic plexuses: the (i) hypogastric nerve (from T12–L3 nerve roots), (ii) pelvic nerves (from sacral nerve roots), and (iii) post-ganglionic fibers from the paravertebral sympathetic thoracolumbar (T12–L3 levels) ganglia chain [25].

A corpus cavernosum is one of a pair of sponge-like tissue of erectile system that contains most of the blood in the penis during an erection [28]. The corpus cavernosum tissue consists of irregular blood-filled spaces lined by endothelium surrounded by a specific type of smooth muscle cells (i.e., cavernosal smooth muscle). Penile tumescence (erection) and detumescence are regulated by a complex process of relaxation and contraction, respectively, of the penile corpus cavernosum that is mediated mainly by the cavernosal nerves [29]. The sympathetic portion of the cavernosal nerve, which is mainly α-adrenergic nerve fibers with norepinephrine as the major transmitter, is widely present in the cavernous trabeculae and surrounding the cavernosal arteries. The sympathetic contraction of cavernosal smooth muscles is responsible for the semicontracted (flaccid) or detumescence state. Additionally, vascular endothelium-derived vasoconstrictors (e.g., endothelin and prostaglandin F_2α_) also contribute to the maintenance of the intracorporeal smooth muscle contraction [25]. On the other hand, the parasympathetic portion of the cavernosal nerve is responsible for the cavernosal smooth muscle relaxation and penile erection. NO as the main non-adrenergic non-cholinergic (NANC) neurotransmitter is released upon the cavernosal nerve stimulation and is the key mediator of cavernosal smooth muscle relaxation [30]. NO is synthesized from endogenous L-arginine by the enzyme neuronal nitric oxide synthase (nNOS) in the cavernosal nerves. Acetylcholine is also released from the cavernosal nerves upon their stimulation, and contributes to the penile erection via two mechanisms: (i) the inhibition of adrenergic neurons, and (ii) stimulation of NO release from the endothelial cells in the corpus cavernosum via activation of the endothelial NOS (eNOS) isoform. It is believed that neurogenic nNOS-mediated NO is responsible for the immediate relaxation of penile vessels and the corpus cavernosum, whereas endothelial eNOS-mediated NO plays a role in maintaining an erection [25,29,31]. NO mediates cavernosal smooth muscle relaxation through the activation of guanylyl cyclase and thereby cyclic guanosine monophosphate (cGMP) production. Besides NO, prostaglandins E_1_ and E_2_ can also be generated by the cyclooxygenase (COX) enzyme in the endothelial cells upon activation with acetylcholine [32]. Other neurotransmitters that may participate in the penile erection are vasoactive intestinal peptide (VIP) [33], calcitonin gene-related peptide (CGRP) [34], and endogenous cannabinoids [35,36].

## 3. Lithium and Sexual Dysfunction

### 3.1. Epidemiology

Reports of lithium’s effects on sexual function dates to an early study in the 1970s [37] when sexual dysfunction was found in 5 out of 33 patients treated with lithium carbonate for bipolar disorder. In two patients, when lithium was substituted with a placebo, erectile dysfunction improved, but re-occurred after resuming lithium carbonate [37,38]. In a subsequent larger study on 50 patients with mood disorders receiving lithium monotherapy, half of the patients reported decreased sexual desire with lithium treatment [39]. The next study in 1982 similarly reported lithium-related sexual dysfunction in two patients with major affective disorder, bipolar type I [40], in one of which there was a rapid disappearance of dysfunction after a blind placebo substitution [40]. In another study on 24 patients with major affective disorders who were given prophylactic lithium treatment, changes in sexual function during lithium treatment were reported by 25% of the patients [41]. Even a larger study on 104 male and female outpatients with a DSM-III diagnosis of bipolar disorder found that combination therapy with lithium and benzodiazepines may increase the rate of sexual dysfunction compared to lithium monotherapy or combined with other drugs (i.e., tricyclic antidepressants, neuroleptics, tryptophan, or carbamazepine) [42]. In another study on 35 bipolar and schizoaffective men receiving lithium monotherapy, sexual dysfunction on at least two items of the sexual function questionnaire was reported in 31.4% of patients, with notable results of reduction in the frequency of sexual thoughts, loss of erection during sex, and difficulties in achieving and maintaining erections (ease of arousal) in 23%, 20%, and 14% of patients, respectively [20]. No statistical correlation was found between sexual function scores and serum lithium levels [20]. A study in Italy on 51 patients with DSM IV-TR bipolar I/II disorder and on long-term lithium monotherapy found significantly less sexual intercourses, sexual fantasies, sexual desires, as well as less pleasure and satisfaction during intercourse in these patients compared to 176 age-matched healthy control subjects, and 30% of patients described their sexual problems in association with the introduction of lithium intake [43]. Another study in India on 100 patients with bipolar disorder receiving lithium (mean treatment duration of ~120 months), using the Arizona Sexual Experience Scale and Brief Adherence Rating Scale to assess sexual function, found that one third of patients had sexual dysfunction [21]. Notably, patients with sexual dysfunction were older, had lower levels of functioning, a higher number of other lithium-related side effects, and poor medication compliance [21]. Finally, a more recent multicenter, cross-sectional study on 114 outpatients with bipolar disorder found that lithium in monotherapy or combined with benzodiazepines is related to more sexual dysfunction, as assessed by the Changes in Sexual Functioning Questionnaire Short Form (CSFQ-14), and worse sexual desire compared to anticonvulsants (valproate or lamotrigine) monotherapy [44]. The addition of benzodiazepines or anticonvulsants to lithium was also found to negatively impact sexual orgasm; however, sexual arousal could improve with the addition of benzodiazepines to lithium [44].

### 3.2. Underlying Mechanisms

#### 3.2.1. Central Mechanisms

There are scarce data on the central mechanisms underlying the effects of lithium on sexual function. The first preclinical study in this regard was published in 1992, when a 30-day chronic administration of lithium chloride (LiCl, 600 mg/L in drinking water) significantly reduced penile erection induced by the subcutaneous (s.c.) administration of the mixed D_1_/D_2_ dopamine receptor agonist apomorphine in male albino rats [45]. Given the fact that apomorphine-induced penile erection is mainly mediated by central dopaminergic neurotransmission [46], especially in the MPOA region [47], these data may provide an evidence that lithium affects erectile function via a central mechanism (Table 1). Consistent with these findings, another study also showed that acute LiCl administration (50 and 100 mg/kg, intraperitoneally [i.p.]) significantly reduced apomorphine-induced penile erections in male Sprague-Dawley rats [48], an effect that was prevented by pretreatment with the NO precursor L-arginine (100 mg/kg, i.p.) or sildenafil (3.5 mg/kg, i.p.) [48]. Additionally, combined sub-effective doses of lithium (30 mg/kg, i.p.) and the non-selective NOS inhibitor *N*^G^-nitro-L-arginine methyl ester (L-NAME, 10 mg/kg, i.p.) exerted a significant inhibition of apomorphine-induced penile erections [48]. Although these findings may suggest a role for nitrergic neurotransmission in this inhibitory effect of lithium on penile erection, it is not clear whether it is related to central or peripheral NO signaling, as both central (e.g., in the hypothalamus) or peripheral (i.e., in the penile cavernosal tissue) NO transmission are involved in penile erection and sexual function. Accordingly, other preclinical studies have shown that lithium can decrease the level of NO metabolites or the conversion of L-arginine to L-citrulline (a byproduct of NO synthesis) in different brain regions including the hippocampus [49,50], amygdala [51], cerebellum [52], and microglia [53].

In another study, the intracerebroventricular (i.c.v.) injection of oxytocin (50 and 200 ng/10 μL) dose-dependently induced yawning and penile erection in male Wistar rats [54], likely through the activation of oxytocinergic neurons in the PVN, as evidenced by an increase in the number of c-Fos positive nuclei in the PVN after an oxytocin injection [54]. Pretreatment with LiCl (0.5 and 1.0 mEq, i.p.) at 15 min prior to the oxytocin injection reduced the oxytocin-induced yawning and penile erection in a dose-dependent manner in these animals [54].

It is well-established that the dopaminergic transmission and dopamine D_1_ (D_1_, D_5_)- and D_2_ (D_2_-D_4_)-like receptors in the PVN, MPO, midbrain tegmental region (near the substantia nigra), spinal cord, and in the erectile tissue overall play facilitatory roles in penile erection [24,25,60,61]. On the other hand, lithium is found to reduce dopamine levels in different brain regions such as the rat striatum [62], rat whole brain [63], rat nucleus accumbens [64,65], and mouse nucleus accumbens [66], although some studies report no alterations [67,68]. Lithium enhances the levels of dopamine D_2_ receptor mRNA coding (but not protein) in the rat nucleus accumbens and striatum [69,70]; however, it hinders the receptor binding [71] and the dopamine-induced, adenylyl cyclase-mediated cAMP accumulation [72]. Therefore, it is possible that the attenuation of dopaminergic transmission by lithium may underlie its side effects on erectile function. Investigating the effects of lithium on dopamine signaling, especially in the PVN and MPO, could be a topic of interest for future studies.

Glutamatergic/NMDA receptor signaling, especially in the PVN, also plays a facilitatory role in sexual and erectile function [24,26]. Lithium attenuates glutamatergic/NMDA receptor signaling in various brain regions [73,74,75], which is thought to be a target for the therapeutic effects of lithium in mood disorders [7]. However, given the role of this signaling pathway in erection, the attenuation of glutamatergic neurotransmission may be another pathway underlying lithium-induced sexual dysfunction.

Serotonin (5-hydroxytryptamine or 5-HT) is another key transmitter in the modulation of penile erection. Serotonergic signaling from the nucleus paragigantocellularis onto the hypothalamus, the limbic system, the neocortex, and the spinal cord plays inhibitory roles in erection [27]. On the other hand, lithium facilitates central serotonergic neurotransmission [9,76], an effect that could be due to either the inhibition of presynaptic 5-HT_1A_ and 5-HT_1B_ autoreceptors [77,78] or alteration of postsynaptic 5-HT_1A_ and 5-HT_2_ receptors in the frontal cortex and hippocampus [79]. These results may raise the possibility that lithium may affect sexual function through the facilitation of central serotonergic transmission.

It is well established that the dysregulation of the hypothalamo-pituitary-adrenal (HPA) axis is involved in the pathogenesis of mood disorders [80] and sexual dysfunction [81]. Overall, glucocorticoids regulation is exerted on hypothalamic corticotropin-releasing factor (CRF) neurons in the PVN [82] and on other central regions, including the hippocampus and amygdala [83]. In a study on male and female Sprague-Dawley rats, a 10-day treatment with LiCl (6 mM/kg/day, i.p.) caused a significant increase in both hypothalamic and hippocampal Type II glucocorticoid receptor mRNA levels [55]. Moreover, hippocampal NO overproduction and HPA axis dysregulation due to juvenile social isolation stress in male mice were found to decrease after acute LiCl (10 mg/kg, i.p.) treatment [50]. Other evidence for possible hormonal changes related to lithium was reported when a significant decrease in the testosterone level and spermatogenesis in male Wistar rats, as well as increase in estradiol level in female Wistar rats, were observed after 28 days of treatment with lithium carbonate (2 or 4 gr/kg food) [56]. Overall, these preclinical data represent different central mechanisms by which lithium therapy can affect sexual and erectile function.

#### 3.2.2. Peripheral Mechanisms

**Figure 2:**
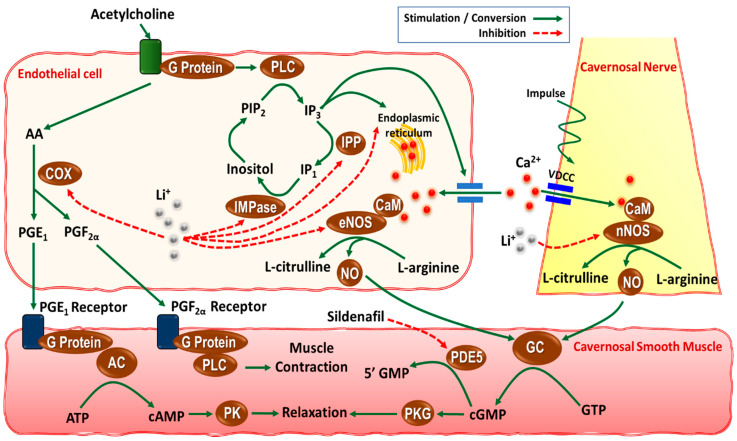
Schematic representation of possible mechanisms underlying the effects of lithium on neurogenic and endothelium-mediated relaxation of cavernosal smooth muscle, and thereby erectile function. In endothelial cells, acetylcholine binds to the G-protein-coupled receptor that can activate phospholipase C (PLC) and thereby increasing the production of inositol 1,4,5-trisphosphate (IP_3_) [92]. IP_3_ releases Ca^2+^ from the endoplasmic reticulum or increases Ca^2+^ influx via calcium channels. Ca^2+^ then binds to calmodulin and activates endothelial nitric oxide (NO) synthase (eNOS) that eventually leads to production of NO from L-arginine. Lithium (Li^+^) can negatively affect this process through several mechanisms. It can inhibit the IP_3_ production cycle via inhibition of inositol monophastase (IMPase) or inositol phosphatase (IPP) [93]. There is also evidence that lithium prevents IP_3_-sensitive Ca^2+^-release from the endoplasmic reticulum [96,97]. Different prostaglandins produced from arachidonic acid (AA) via cyclooxygenase (COX) activity also contribute to both cavernosal smooth muscle contraction and relaxation. Prostaglandins E_1_ (PGE_1_) and PGE_2_ activate adenylyl cyclase (AC) and increase the production of cyclic adenosine monophosphate (cAMP), which ultimately leads to muscle relaxation. However, prostaglandin F_2α_ (PGF_2α_) causes muscle contraction via activation of PLC. Lithium is reported to decrease COX-2 expression and PGE_2_ level in rat brain [101,102]. Neurogenic relaxation of the cavernosal smooth muscle is also mediated by neuronal NO, generated by the neuronal NOS (nNOS) activity in the cavernosal nerves [35]. Lithium can also decrease eNOS and nNOS activities as evidenced by decreased eNOS expression in vascular tissues as well as NO metabolites in brain tissues [51,52,98]. ATP, adenosine triphosphate; CaM, calmodulin; cGMP, cyclic guanosine monophosphate; GC, guanylyl cyclase; GTP, guanosine triphosphate; PK, protein kinase; PKG, protein kinase G; PIP_2_, phosphoinositide 4,5-biphosphate; VDCC, voltage-dependent calcium channel.

Besides the possible central effects of lithium on sexual and erectile function, there is evidence that lithium may directly affect the function of penile cavernosal tissue, and thereby erectile function [32,57,58,59]. The first preclinical evidence was published in 2007 when Sadeghipour et al. [57] examined the effects of the acute administration of lithium in vitro on both neurogenic and endothelium-mediated relaxation of rat isolated corpus cavernosum. In this study, LiCl (0.5, 1, and 5 mM) significantly decreased both neurogenic NANC and endothelium-mediated cavernosal smooth muscle relaxation in a concentration-dependent manner [57]. Notably, combined sub-effective concentrations of LiCl (0.3 mM) and L-NAME also exerted a significant inhibition of both the neurogenic and endothelial relaxation of corpus cavernosum. On the other hand, L-arginine prevented such effects of lithium [57]. Therefore, it was suggested that both endothelium- and NANC-mediated NO is involved in the inhibitory effects of lithium on the rat cavernosal smooth muscle relaxation, and thereby penile erection [57] (Figure 2). The NO-mediated NANC relaxation in other rat tissues such as the gastric fundus [84] and anococcygeus muscle [85] were also found to be attenuated with acute lithium treatment in vitro. Such acute effects of lithium were also evaluated in guinea pig isolated corpus cavernosum in vitro [59]. LiCl (0.5, 1, and 5 mM) similarly decreased endothelium-mediated cavernosal smooth muscle relaxation isolated from guinea pigs; however, it did not alter neurogenic NANC relaxation [59], a difference that might be due to the differences in the animal species used.

Subsequent studies also found that a 30-day chronic LiCl administration (600 mg/L in drinking water) inhibited both the neurogenic [58] and endothelium-mediated [32] relaxation of rat isolated corpus cavernosum, an adverse effect that was again exacerbated with L-NAME and improved with L-arginine [32,58]. In none of these studies, neither acute nor chronic lithium therapy affected relaxant responses to NO donors and guanylyl cyclase activators, suggesting that NO-mediated cGMP signaling seems unlikely to participate in the effects of lithium on cavernosal smooth muscle relaxation [32,57,58,59].

Acetylcholine-induced, endothelium-dependent vascular relaxation has been shown to be affected by lithium in a variety of other tissues, including vessel grafts from de-nerved murine aortas and porcine middle cerebral arteries [86], rat mesenteric vascular bed [87,88], and rat isolated aorta [89,90]. Endothelial NO signaling was suggested to contribute to the lithium’s effects on endothelium-mediated relaxation in these tissues [86,87,88,89,90]. Acute lithium administration can also inhibit substance P and vasoactive intestinal peptide-induced relaxations of isolated porcine ophthalmic artery in vitro [91].

In endothelial cells, messenger molecules such as acetylcholine binds to the G-protein-coupled receptor that can activate phospholipase C, thereby increasing the production of inositol 1,4,5-trisphosphate (IP_3_) (Figure 2) [92]. IP_3_ releases Ca^2+^ sequestered in the endoplasmic reticulum or enhance Ca^2+^ influx via calcium channels. Ca^2+^ then binds to calmodulin and activates eNOS that eventually leads to the production of NO from L-arginine. The neurogenic relaxation of the cavernosal smooth muscle is also mediated by neuronal NO, as the NANC transmitter, generated by the nNOS activity [35]. Lithium may affect these processes via several mechanisms, including (Figure 2): (i) inhibitory effects on inositol cycle and depletion of inositol via inhibiting inositol phosphatases [93], (ii) decreasing IP_3_-induced Ca^2+^ current from the endoplasmic reticulum (ER) to the intracellular space and reducing ER Ca^2+^ stores [94,95,96,97], (iii) possible inhibitory effects on either eNOS or nNOS activity as evidenced by lithium-induced decreased NO metabolites or L-citrulline in various tissues including blood as well as decreased eNOS expression in human umbilical vein endothelial cells [13,51,52,98].

A notable finding was that in vitro indomethacin (a COX inhibitor) administration was also able to improve the endothelium-mediated cavernosal relaxation in chronically lithium-treated rats compared to control animals [32]. It is well known that endothelium-mediated relaxation in response to acetylcholine results in the production of vasorelaxant prostaglandins (e.g., E_1_ and E_2_) via the COX enzyme activity [25,99,100]. Prostaglandins E_1_ and E_2_ activate adenylyl cyclase (AC) and increase the production of cyclic adenosine monophosphate (cAMP), which ultimately leads to muscle relaxation. On the other hand, vascular endothelium-derived vasoconstrictors such as prostaglandin F_2α_ can also be produced by the activity of COX [25]. Therefore, it is possible that lithium may have caused an imbalance in the prostaglandins production with more of prostaglandin F_2α_ rather than E_1_ or E_2_ in response to acetylcholine, causing an impaired endothelial relaxation response; and indomethacin via inhibiting COX enzyme could prevent such effect of lithium. The serum lithium level was 0.33 mM in chronically treated rats, indicating that these lithium’s adverse effects could occur even at subtherapeutic serum concentrations [32]. In agreement with this assumption, it was shown that chronic lithium treatment (chow containing 1.70 g LiCl per kg for 4 weeks followed by chow containing 2.55 g LiCl per kg for 2 weeks) decreased COX-2 expression and prostaglandin E_2_ concentration in the rat brain [101,102]. Another study found that indomethacin potentiated the acetylcholine-induced relaxation of the isolated aorta from chronic lithium-treated rats [89]. Lithium was also found to decrease cAMP levels in the rat cortex [103] and human umbilical vein endothelial cells [98].

### 3.3. Therapeutic Approaches

Up to date, there is only one published clinical trial to directly address sexual dysfunction related to lithium therapy [104]. In this randomized, double-blinded, placebo-controlled trial on 32 male patients with stable bipolar disorder, it was found that aspirin (240 mg/kg) therapy for 6 weeks significantly improved lithium-related erectile dysfunction compared to placebo, as measured by the International Index for Erectile Function (IIEF) [104]. These results are consistent with the above-mentioned preclinical study in which indomethacin was found to improve chronic lithium-induced impaired endothelium-mediated relaxation of the rat isolated corpus cavernosum [32]. Increased intracellular cGMP is essential for cavernosal smooth muscle relaxation and penile erection (Figure 2); various phosphodiesterases (PDEs), especially PDE type 5 (PDE5), degrade cGMP to 5′-GMP, thereby decreasing its intracellular concentration and preventing adequate erection. PDE5 inhibitors (e.g., sildenafil) have been shown to be beneficial in the treatment of erectile dysfunction related to antipsychotics therapy [105,106]. More recent investigation has also demonstrated that patients treated with lithium combined with other psychotherapeutic agents had improvement in their erectile dysfunction after treatment with PDE5 inhibitors [107]. Clearly, more studies are needed to investigate several other approaches that could be beneficial in improving erectile and sexual function in patients receiving lithium. For instance, based on the above animal studies, L-arginine could be another potential option, as it was able to improve both endothelium- and NANC-mediated cavernosal relaxation in lithium-treated animals [32,48,58,59].

## 4. Conclusions and Perspectives

Despite the fact that lithium has been widely used over the last several decades especially for patients with mood disorders, it may have some adverse effects that may play an important role in patients’ non-compliance. Based on our literature review, a considerable number of patients receiving lithium as monotherapy or combined with other medications, especially benzodiazepines, may experience various symptoms of sexual and erectile dysfunction. The etiology of these adverse effects related to lithium therapy is still not completely understood. However, both central and peripheral mechanisms may play a role. Lithium may affect the function of brain regions involved in sexual function, such as the hypothalamus and penile tissue directly, as evidenced by preclinical studies on rodents and guinea pigs. Understanding the pathologic pathways that are involved in such side effects of lithium can open new avenues for therapeutics in this regard. For instance, consistent with beneficial effects of indomethacin in preclinical studies on lithium-related erectile dysfunction in rats [32], a recent clinical trial found that aspirin may improve erectile function in patients with bipolar disorders taking lithium [104]. Treatment with PDE5 inhibitors could also be another option, as they have been found to be beneficial both in antipsychotic-induced erectile dysfunction [105,106] as well as in subjects treated with lithium combined with other psychotherapeutic agents [107].

## Figures and Tables

**Table 1:** Effects of lithium on erectile function in preclinical studies.

Lithium Treatment	Species	Measurement	Result	Ref
600 mg/L in drinking water, 30 days	Male albino rats	Apomorphine (s.c.)-induced penile erection	↓	[45]
0.5 and 1.0 mEq, i.p., 15 min prior oxytocin	Male Wistar rats	Oxytocin (i.c.v.)-induced penile erection & yawning	↓	[54]
5 to 100 mg/kg, i.p., 30 min prior apomorphine	Male Sprague-Dawley rats	Apomorphine (s.c.)-induced penile erection	↓ (at 50 & 100 mg/kg)	[48]
6 mM/kg/day, i.p., 10 days	Male & female Sprague-Dawley rats	hypothalamic & hippocampal Type II glucocorticoid receptor mRNA levels	↑	[55]
2 or 4 gr/kg food, 28 days	Male Wistar rats	Serum testosterone level & spermatogenesis	↓	[56]
	Female Wistar rats	Serum estradiol level	↑	
0.5, 1, & 5 mM, in vitro, 45 min incubation	Male Sprague-Dawley rats	Neurogenic & endothelium (Ach)-mediated isolated cavernosal muscle relaxation	↓	[57]
600 mg/L in drinking water, 30 days	Male Sprague-Dawley rats	Endothelium (Ach)-mediated isolated cavernosal muscle relaxation	↓	[32]
600 mg/L in drinking water, 30 days	Male Sprague-Dawley rats	Neurogenic isolated cavernosal muscle relaxation	↓	[58]
0.5, 1, & 5 mM, in vitro, 45 min incubation	Male guinea pigs	Endothelium (Ach)-mediated isolated cavernosal muscle relaxation	↓	[59]
		Neurogenic isolated cavernosal muscle relaxation	↔	

Ach, acetylcholine; i.c.v., intracerebroventricular injection; s.c., subcutaneous. ↓, decrease; ↑, increase; ↔, no change.
